# 2-(3,4-Dimeth­oxy­phen­yl)-4,5-diphenyl-1-(prop-2-en-1-yl)-1*H*-imidazole

**DOI:** 10.1107/S1600536812039566

**Published:** 2012-09-22

**Authors:** Shaaban K. Mohamed, Mehmet Akkurt, Frank R. Fronczek, Adel A. E. Marzouk, Antar A. Abdelhamid

**Affiliations:** a Chemistry and Environmental Division, Manchester Metropolitan University, Manchester M1 5GD, England; b Department of Physics, Faculty of Sciences, Erciyes University, 38039 Kayseri, Turkey; c Department of Chemistry, Louisiana State University, Baton Rouge, LA 70803-1804, USA; d Pharmaceutical Chemistry Department, Faculty of Pharmacy, Al Azhar University, Egypt

## Abstract

In the title compound, C_26_H_24_N_2_O_2_, the planar 1*H*-imidazole ring makes dihedral angles of 35.78 (4), 26.35 (5) and 69.75 (5)°, respectively, with the dimeth­oxy­phenyl ring and the phenyl rings in the 4- and 5-positions. In the crystal, C—H⋯O hydrogen bonds connect neighbouring mol­ecules, forming infinite chains running along the *b* axis. Furthermore, the crystal structure exhibits a C—H-⋯π inter­action between a methyl H atom and a phenyl ring from an adjacent mol­ecule.

## Related literature

For the synthesis of imidazole compounds, see: Shalini *et al.* (2010 ▶). For the medicinal properties of imidazole derivatives, see: Adams *et al.* (2001 ▶); Nakamura *et al.* (2004 ▶); Venkatesan *et al.* (2008 ▶); Nanterment *et al.* (2004 ▶); Roman *et al.* (2007 ▶); Congiu *et al.* (2008 ▶). For standard bond distances, see: Allen *et al.* (1987 ▶).
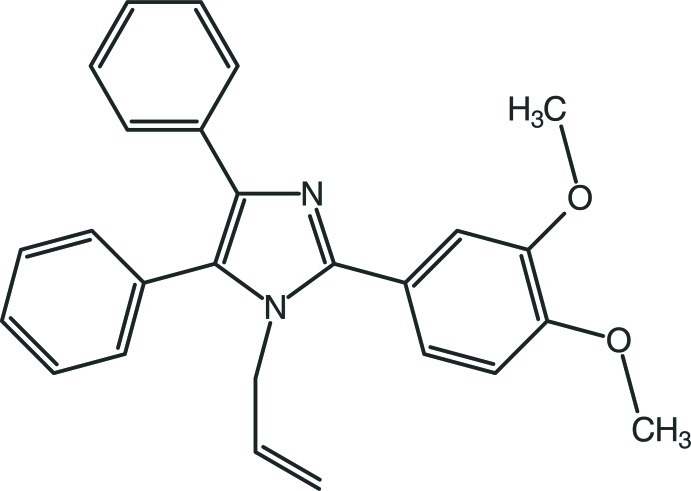


## Experimental

### 

#### Crystal data

C_26_H_24_N_2_O_2_
*M* *_r_* = 396.47
Triclinic, 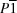

*a* = 8.9683 (4) Å
*b* = 10.7916 (5) Å
*c* = 11.7219 (5) Å
α = 110.174 (2)°
β = 106.267 (2)°
γ = 91.991 (3)°
*V* = 1011.32 (8) Å^3^
*Z* = 2
Mo *K*α radiation
μ = 0.08 mm^−1^
*T* = 90 K
0.36 × 0.12 × 0.06 mm

#### Data collection

Bruker Kappa APEXII DUO diffractometer
Absorption correction: multi-scan (*SADABS*; Sheldrick, 2004 ▶) *T*_min_ = 0.971, *T*_max_ = 0.995
17346 measured reflections
6149 independent reflections
4408 reflections with *I* > 2σ(*I*)
*R*_int_ = 0.039
Standard reflections: 0

#### Refinement

*R*[*F* ^2^ > 2σ(*F* ^2^)] = 0.048
*wR*(*F* ^2^) = 0.127
*S* = 1.02
6149 reflections
273 parameters
H-atom parameters constrained
Δρ_max_ = 0.46 e Å^−3^
Δρ_min_ = −0.25 e Å^−3^

### 

Data collection: *APEX2* (Bruker, 2005 ▶); cell refinement: *SAINT* (Bruker, 2005 ▶); data reduction: *SAINT*; program(s) used to solve structure: *SIR97* (Altomare *et al.*, 1999 ▶); program(s) used to refine structure: *SHELXL97* (Sheldrick, 2008 ▶); molecular graphics: *ORTEP-3 for Windows* (Farrugia, 1997 ▶) and *PLATON* (Spek, 2009 ▶); software used to prepare material for publication: *WinGX* (Farrugia, 1999 ▶) and *PARST* (Nardelli, 1983 ▶).

## Supplementary Material

Crystal structure: contains datablock(s) global, I. DOI: 10.1107/S1600536812039566/bt6835sup1.cif

Structure factors: contains datablock(s) I. DOI: 10.1107/S1600536812039566/bt6835Isup2.hkl

Supplementary material file. DOI: 10.1107/S1600536812039566/bt6835Isup3.cml

Additional supplementary materials:  crystallographic information; 3D view; checkCIF report

## Figures and Tables

**Table 1:** Hydrogen-bond geometry (Å, °) *Cg*3 is the centroid of the C15–C20 phenyl ring.

*D*—H⋯*A*	*D*—H	H⋯*A*	*D*⋯*A*	*D*—H⋯*A*
C7—H9*B*⋯O1^i^	0.96	2.57	3.515 (2)	170
C8—H8*B*⋯*Cg*3^ii^	0.96	2.98	3.8316 (17)	149

Symmetry codes: (i) 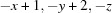; (ii) 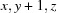.

**Fig. 1.**
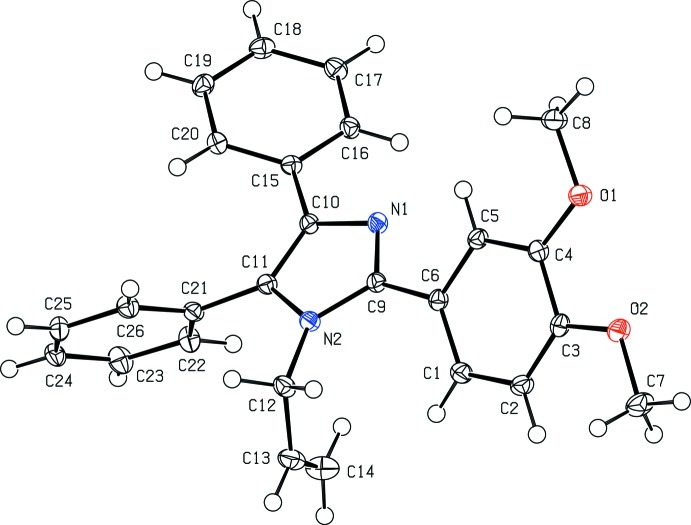
The molecular structure of (I) with the atom numbering scheme. Displacement ellipsoids for non-H atoms are drawn at the 50% probability level.

**Fig. 2.**
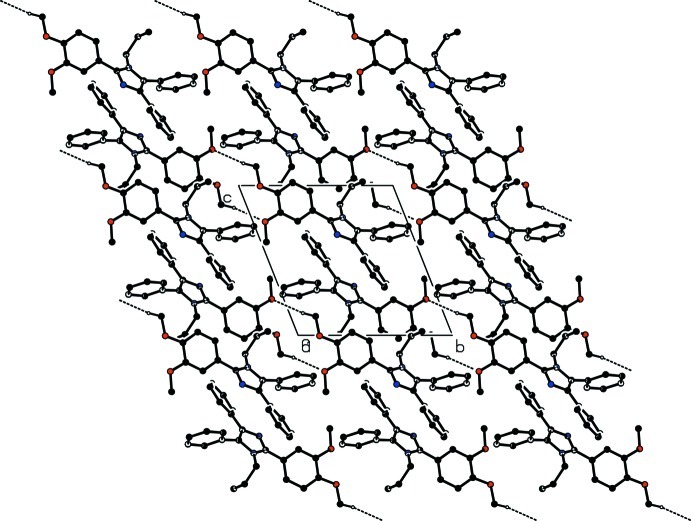
View of the crystal packing and hydrogen bonding of (I) down the a axis. H atoms not involved in hydrogen bonds have been omitted for clarity.

**Fig. 3.**
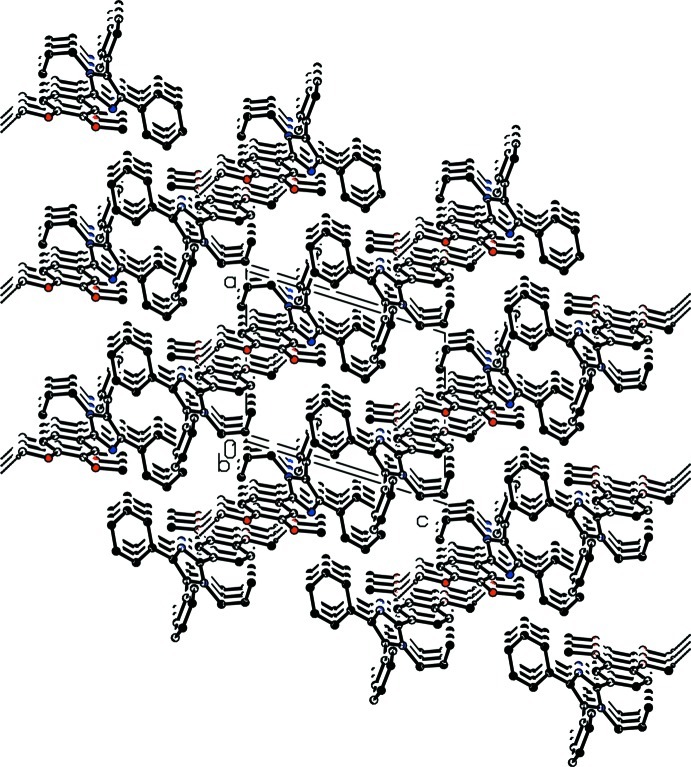
View of the crystal packing and hydrogen bonding of (I) down the b axis. H atoms not involved in hydrogen bonds have been omitted for clarity.

## supplementary crystallographic information

### Comment

The high therapeutic properties of the imidazole related drugs have encouraged the medicinal chemists to synthesize a large number of novel chemotherapeutic agents incorporating the imidazole nucleus (Shalini *et al.*, 2010). The broad medicinal properties of imidazole drugs include anticancer, β-lactamase inhibitors, 20-HETE (20-hydroxy-5,8,11,14-eicosatetraenoic acid) synthase inhibitors, carboxypeptidase inhibitors, hemeoxygenase inhibitors, anti-aging agents, anticoagulants, anti-inflammatory, antibacterial, antifungal, antiviral, anti-tubercular, anti-diabetic and antimalarial (Congiu *et al.*, 2008; Venkatesan *et al.*, 2008; Nakamura *et al.*, 2004; Roman *et al.*, 2007; Nanterment *et al.*, 2004 and Adams *et al.*, 2001). In this respect and in continuation of our on-going study for synthesis of bioactive molecules, we herein report synthesis and crystal structure of the title compound (I) among series of other imidazole derivatives.

The molecular structure of (I), (Fig. 1), has not a planar conformation. The (N1/N2/C9—C11) 1*H*-imidazole ring which is planar [maximum deviation = 0.005 (1) Å for C11] forms dihedral angles of 35.78 (4), 26.35 (5) and 69.75 (5)°, respectively, with the C1–C6 benzene ring and the C15–20 and C21–C26 phenyl rings. The plane of the allyl group makes a dihedral angle of 82.09 (12)° with the plane of the 1*H*-imidazole ring.

In (I), all bond lengths and bond angles are within normal range (Allen *et al.*, 1987). The C3–C4–O1–C8 and C4–C3–O2–C7 torsion angles are 171.98 (13) and -176.30 (13)°, respectively.

In the crystal packing, molecules are linked by C—H···O hydrogen bonds, forming infinite chains running along the *b* axis (Table 1, Figs. 2 & 3). In addition, a C–H···π interaction is observed between the (C8)H8B methyl H atom and the C15–C20 phenyl ring of the adjacent molecule (Table 1).

### Experimental

A mixture of 25 ml. of dimethyl sulfoxide and 2.4 g. (40 mmol) of potassium hydroxide was added in a 50-ml. volumetric flask equipped with a magnetic stirring bar. The mixture was stirred at room temperature for 5 minutes before adding of 3.26 g. (10 mmole) of 2-(3,4-dimethoxyphenyl)-4,5-diphenyl-1*H*-imidazole. Stirring was continued for 45 minutes, then 4.80 g. (20 mmol) of allylbromide was added. After being stirred for an additional 45 minutes the mixture was diluted with 20 ml. of water. The organic product was extracted with three 20-ml. portions of diethyl ether, and each ether layer was washed with three 10-ml. portions of water. The combined ether layers were dried over calcium chloride, and the solvent was removed at slightly reduced pressure. The excess allyl bromide was removed by distillation at approximately 15 mm. The residue was solidified upon cooling and scratching to furnish the title compound (3.22 g; 88%). Mono crystals suitable for X-ray analyses were obtained by slow evaporation method from ethanol at room temperature. *M*.p. 486 – 488 K.

### Refinement

Hydrogen atoms were located geometrically and refined using a riding model with C—H = 0.93–0.97 Å, and with *U*_iso_ =1.2*U*_eq_(C) or 1.5*U*_eq_(C_methyl_). The methyl groups were allowed to rotate but not to tip.

### Crystal data

**Table:**

C_26_H_24_N_2_O_2_	*Z* = 2
*M**_r_* = 396.47	*F*(000) = 420
Triclinic, *P*1	*D*_x_ = 1.302 Mg m^−^^3^
Hall symbol: -P 1	Mo *K*α radiation, λ = 0.71073 Å
*a* = 8.9683 (4) Å	Cell parameters from 3877 reflections
*b* = 10.7916 (5) Å	θ = 2.2–30.5°
*c* = 11.7219 (5) Å	µ = 0.08 mm^−^^1^
α = 110.174 (2)°	*T* = 90 K
β = 106.267 (2)°	Needle, colourless
γ = 91.991 (3)°	0.36 × 0.12 × 0.06 mm
*V* = 1011.32 (8) Å^3^	

### Data collection

**Table:**

Bruker Kappa APEXII DUO diffractometer	6149 independent reflections
Radiation source: sealed tube	4408 reflections with *I* > 2σ(*I*)
TRIUMPH curved graphite monochromator	*R*_int_ = 0.039
φ and ω scans	θ_max_ = 30.5°, θ_min_ = 2.0°
Absorption correction: multi-scan (*SADABS*; Sheldrick, 2004)	*h* = −12→12
*T*_min_ = 0.971, *T*_max_ = 0.995	*k* = −15→15
17346 measured reflections	*l* = −16→16

### Refinement

**Table:**

Refinement on *F*^2^	Primary atom site location: structure-invariant direct methods
Least-squares matrix: full	Secondary atom site location: difference Fourier map
*R*[*F*^2^ > 2σ(*F*^2^)] = 0.048	Hydrogen site location: inferred from neighbouring sites
*wR*(*F*^2^) = 0.127	H-atom parameters constrained
*S* = 1.02	*w* = 1/[σ^2^(*F*_o_^2^) + (0.0575*P*)^2^ + 0.309*P*] where *P* = (*F*_o_^2^ + 2*F*_c_^2^)/3
6149 reflections	(Δ/σ)_max_ < 0.001
273 parameters	Δρ_max_ = 0.46 e Å^−^^3^
0 restraints	Δρ_min_ = −0.25 e Å^−^^3^

### Special details

**Table:**

Geometry. Bond distances, angles *etc*. have been calculated using the rounded fractional coordinates. All su's are estimated from the variances of the (full) variance-covariance matrix. The cell e.s.d.'s are taken into account in the estimation of distances, angles and torsion angles
Refinement. Refinement on *F*^2^ for ALL reflections except those flagged by the user for potential systematic errors. Weighted *R*-factors *wR* and all goodnesses of fit *S* are based on *F*^2^, conventional *R*-factors *R* are based on *F*, with *F* set to zero for negative *F*^2^. The observed criterion of *F*^2^ > σ(*F*^2^) is used only for calculating -*R*-factor-obs *etc*. and is not relevant to the choice of reflections for refinement. *R*-factors based on *F*^2^ are statistically about twice as large as those based on *F*, and *R*-factors based on ALL data will be even larger.

### Fractional atomic coordinates and isotropic or equivalent isotropic displacement parameters (Å^2^)

**Table:**

	*x*	*y*	*z*	*U*_iso_*/*U*_eq_	
O1	0.60501 (12)	0.92104 (10)	0.24135 (9)	0.0174 (3)	
O2	0.57242 (12)	0.86263 (10)	0.00369 (9)	0.0180 (3)	
N1	0.72481 (13)	0.48753 (11)	0.32705 (10)	0.0136 (3)	
N2	0.88572 (13)	0.40893 (11)	0.21328 (10)	0.0135 (3)	
C1	0.70264 (16)	0.55194 (14)	0.03650 (12)	0.0160 (4)	
C2	0.65044 (16)	0.64264 (14)	−0.02155 (12)	0.0163 (4)	
C3	0.62078 (15)	0.76560 (13)	0.04910 (12)	0.0144 (3)	
C4	0.63977 (16)	0.79788 (13)	0.18038 (12)	0.0141 (3)	
C5	0.69306 (15)	0.70881 (13)	0.23773 (12)	0.0138 (3)	
C6	0.72605 (15)	0.58461 (13)	0.16638 (12)	0.0134 (3)	
C7	0.5608 (2)	0.83768 (15)	−0.12690 (13)	0.0229 (4)	
C8	0.64201 (19)	0.96524 (14)	0.37715 (13)	0.0205 (4)	
C9	0.77680 (15)	0.49333 (13)	0.23350 (12)	0.0129 (3)	
C10	0.80286 (15)	0.39533 (13)	0.36895 (12)	0.0125 (3)	
C11	0.90182 (15)	0.34498 (13)	0.29931 (12)	0.0129 (3)	
C12	0.97719 (16)	0.39151 (14)	0.12516 (13)	0.0162 (4)	
C13	0.91694 (18)	0.26993 (15)	0.00627 (13)	0.0207 (4)	
C14	0.7876 (2)	0.18741 (17)	−0.02524 (15)	0.0277 (5)	
C15	0.77327 (15)	0.36358 (12)	0.47378 (12)	0.0127 (3)	
C16	0.62980 (16)	0.38237 (13)	0.49728 (12)	0.0146 (3)	
C17	0.59989 (16)	0.36083 (14)	0.59963 (13)	0.0167 (4)	
C18	0.71297 (17)	0.31669 (14)	0.67889 (13)	0.0172 (4)	
C19	0.85580 (17)	0.29638 (14)	0.65568 (13)	0.0167 (4)	
C20	0.88689 (16)	0.32016 (13)	0.55478 (12)	0.0150 (3)	
C21	0.99892 (16)	0.23750 (13)	0.30191 (12)	0.0139 (3)	
C22	0.92335 (17)	0.10790 (14)	0.26220 (14)	0.0180 (4)	
C23	1.00989 (18)	0.00593 (14)	0.27258 (14)	0.0207 (4)	
C24	1.17247 (17)	0.03201 (14)	0.32038 (13)	0.0181 (4)	
C25	1.24860 (16)	0.16004 (14)	0.35776 (13)	0.0175 (4)	
C26	1.16261 (16)	0.26308 (14)	0.34948 (13)	0.0162 (3)	
H1	0.72180	0.46930	−0.01200	0.0190*	
H2	0.63550	0.62000	−0.10840	0.0200*	
H5	0.70730	0.73110	0.32450	0.0170*	
H8A	0.57790	0.90890	0.39830	0.0310*	
H8B	0.62230	1.05560	0.41010	0.0310*	
H8C	0.75070	0.96090	0.41430	0.0310*	
H9A	0.66190	0.82570	−0.13840	0.0340*	
H9B	0.52540	0.91210	−0.14830	0.0340*	
H9C	0.48740	0.75850	−0.18160	0.0340*	
H12A	1.08480	0.38680	0.16900	0.0190*	
H12B	0.97800	0.46930	0.10170	0.0190*	
H13	0.97540	0.25080	−0.05030	0.0250*	
H14A	0.72580	0.20300	0.02870	0.0330*	
H14B	0.75810	0.11360	−0.10140	0.0330*	
H16	0.55270	0.40990	0.44340	0.0170*	
H17	0.50460	0.37590	0.61490	0.0200*	
H18	0.69340	0.30080	0.74690	0.0210*	
H19	0.93150	0.26650	0.70840	0.0200*	
H20	0.98340	0.30720	0.54110	0.0180*	
H22	0.81450	0.08960	0.22860	0.0220*	
H23	0.95870	−0.08010	0.24740	0.0250*	
H24	1.23020	−0.03640	0.32730	0.0220*	
H25	1.35770	0.17720	0.38850	0.0210*	
H26	1.21420	0.34920	0.37570	0.0190*	

### Atomic displacement parameters (Å^2^)

**Table:**

	*U*^11^	*U*^22^	*U*^33^	*U*^12^	*U*^13^	*U*^23^
O1	0.0249 (5)	0.0137 (5)	0.0147 (4)	0.0065 (4)	0.0063 (4)	0.0060 (4)
O2	0.0244 (5)	0.0182 (5)	0.0155 (4)	0.0069 (4)	0.0064 (4)	0.0108 (4)
N1	0.0149 (5)	0.0132 (5)	0.0146 (5)	0.0035 (4)	0.0050 (4)	0.0068 (4)
N2	0.0159 (5)	0.0133 (5)	0.0135 (5)	0.0036 (4)	0.0061 (4)	0.0064 (4)
C1	0.0183 (7)	0.0148 (6)	0.0152 (6)	0.0045 (5)	0.0061 (5)	0.0052 (5)
C2	0.0192 (7)	0.0179 (7)	0.0128 (6)	0.0030 (5)	0.0054 (5)	0.0064 (5)
C3	0.0138 (6)	0.0155 (6)	0.0155 (6)	0.0023 (5)	0.0034 (5)	0.0085 (5)
C4	0.0143 (6)	0.0132 (6)	0.0154 (6)	0.0022 (5)	0.0048 (5)	0.0057 (5)
C5	0.0146 (6)	0.0154 (6)	0.0126 (5)	0.0024 (5)	0.0039 (5)	0.0067 (5)
C6	0.0132 (6)	0.0140 (6)	0.0144 (6)	0.0024 (5)	0.0042 (5)	0.0069 (5)
C7	0.0328 (8)	0.0236 (8)	0.0159 (6)	0.0058 (6)	0.0071 (6)	0.0117 (6)
C8	0.0295 (8)	0.0180 (7)	0.0150 (6)	0.0078 (6)	0.0081 (6)	0.0058 (5)
C9	0.0130 (6)	0.0123 (6)	0.0130 (5)	0.0026 (5)	0.0035 (5)	0.0047 (5)
C10	0.0134 (6)	0.0113 (6)	0.0132 (5)	0.0024 (5)	0.0039 (5)	0.0050 (5)
C11	0.0144 (6)	0.0112 (6)	0.0133 (5)	0.0022 (5)	0.0043 (5)	0.0046 (5)
C12	0.0178 (6)	0.0171 (7)	0.0176 (6)	0.0054 (5)	0.0094 (5)	0.0078 (5)
C13	0.0272 (8)	0.0202 (7)	0.0177 (6)	0.0065 (6)	0.0123 (6)	0.0063 (6)
C14	0.0337 (9)	0.0261 (8)	0.0203 (7)	0.0011 (7)	0.0109 (7)	0.0032 (6)
C15	0.0153 (6)	0.0100 (6)	0.0128 (5)	0.0015 (5)	0.0047 (5)	0.0041 (5)
C16	0.0157 (6)	0.0138 (6)	0.0158 (6)	0.0043 (5)	0.0056 (5)	0.0067 (5)
C17	0.0171 (6)	0.0170 (7)	0.0184 (6)	0.0033 (5)	0.0086 (5)	0.0071 (5)
C18	0.0230 (7)	0.0157 (6)	0.0146 (6)	0.0016 (5)	0.0070 (5)	0.0069 (5)
C19	0.0197 (7)	0.0150 (6)	0.0150 (6)	0.0033 (5)	0.0030 (5)	0.0070 (5)
C20	0.0148 (6)	0.0145 (6)	0.0164 (6)	0.0038 (5)	0.0052 (5)	0.0060 (5)
C21	0.0168 (6)	0.0133 (6)	0.0134 (5)	0.0048 (5)	0.0064 (5)	0.0056 (5)
C22	0.0151 (6)	0.0157 (7)	0.0229 (7)	0.0029 (5)	0.0051 (5)	0.0072 (6)
C23	0.0237 (7)	0.0124 (6)	0.0264 (7)	0.0040 (6)	0.0082 (6)	0.0073 (6)
C24	0.0219 (7)	0.0167 (7)	0.0187 (6)	0.0097 (6)	0.0084 (5)	0.0079 (5)
C25	0.0143 (6)	0.0200 (7)	0.0188 (6)	0.0053 (5)	0.0045 (5)	0.0083 (6)
C26	0.0170 (6)	0.0134 (6)	0.0175 (6)	0.0022 (5)	0.0053 (5)	0.0051 (5)

### Geometric parameters (Å, º)

**Table:**

O1—C4	1.3685 (18)	C22—C23	1.388 (2)
O1—C8	1.4286 (17)	C23—C24	1.387 (2)
O2—C3	1.3618 (18)	C24—C25	1.384 (2)
O2—C7	1.4321 (17)	C25—C26	1.390 (2)
N1—C9	1.3250 (18)	C1—H1	0.9300
N1—C10	1.3835 (19)	C2—H2	0.9300
N2—C9	1.3721 (19)	C5—H5	0.9300
N2—C11	1.3854 (18)	C7—H9A	0.9600
N2—C12	1.4583 (18)	C7—H9B	0.9600
C1—C2	1.398 (2)	C7—H9C	0.9600
C1—C6	1.3899 (18)	C8—H8A	0.9600
C2—C3	1.381 (2)	C8—H8B	0.9600
C3—C4	1.4134 (18)	C8—H8C	0.9600
C4—C5	1.379 (2)	C12—H12A	0.9700
C5—C6	1.406 (2)	C12—H12B	0.9700
C6—C9	1.470 (2)	C13—H13	0.9300
C10—C11	1.3721 (19)	C14—H14A	0.9300
C10—C15	1.4712 (19)	C14—H14B	0.9300
C11—C21	1.478 (2)	C16—H16	0.9300
C12—C13	1.491 (2)	C17—H17	0.9300
C13—C14	1.318 (3)	C18—H18	0.9300
C15—C16	1.397 (2)	C19—H19	0.9300
C15—C20	1.3991 (19)	C20—H20	0.9300
C16—C17	1.391 (2)	C22—H22	0.9300
C17—C18	1.389 (2)	C23—H23	0.9300
C18—C19	1.392 (2)	C24—H24	0.9300
C19—C20	1.390 (2)	C25—H25	0.9300
C21—C22	1.393 (2)	C26—H26	0.9300
C21—C26	1.396 (2)		
			
C4—O1—C8	116.86 (11)	C1—C2—H2	120.00
C3—O2—C7	116.81 (12)	C3—C2—H2	120.00
C9—N1—C10	105.70 (12)	C4—C5—H5	120.00
C9—N2—C11	106.90 (11)	C6—C5—H5	120.00
C9—N2—C12	128.55 (12)	O2—C7—H9A	109.00
C11—N2—C12	124.46 (12)	O2—C7—H9B	109.00
C2—C1—C6	120.37 (13)	O2—C7—H9C	110.00
C1—C2—C3	120.52 (12)	H9A—C7—H9B	109.00
O2—C3—C2	125.76 (12)	H9A—C7—H9C	109.00
O2—C3—C4	114.84 (12)	H9B—C7—H9C	109.00
C2—C3—C4	119.40 (13)	O1—C8—H8A	109.00
O1—C4—C3	114.91 (12)	O1—C8—H8B	109.00
O1—C4—C5	125.16 (12)	O1—C8—H8C	109.00
C3—C4—C5	119.92 (13)	H8A—C8—H8B	110.00
C4—C5—C6	120.68 (12)	H8A—C8—H8C	109.00
C1—C6—C5	119.06 (13)	H8B—C8—H8C	109.00
C1—C6—C9	123.53 (13)	N2—C12—H12A	109.00
C5—C6—C9	117.34 (11)	N2—C12—H12B	109.00
N1—C9—N2	111.38 (12)	C13—C12—H12A	109.00
N1—C9—C6	122.61 (13)	C13—C12—H12B	109.00
N2—C9—C6	125.96 (12)	H12A—C12—H12B	108.00
N1—C10—C11	110.18 (12)	C12—C13—H13	117.00
N1—C10—C15	120.50 (12)	C14—C13—H13	117.00
C11—C10—C15	129.32 (13)	C13—C14—H14A	120.00
N2—C11—C10	105.85 (12)	C13—C14—H14B	120.00
N2—C11—C21	124.07 (12)	H14A—C14—H14B	120.00
C10—C11—C21	129.91 (13)	C15—C16—H16	119.00
N2—C12—C13	113.96 (13)	C17—C16—H16	119.00
C12—C13—C14	125.34 (15)	C16—C17—H17	120.00
C10—C15—C16	119.34 (12)	C18—C17—H17	120.00
C10—C15—C20	122.15 (13)	C17—C18—H18	120.00
C16—C15—C20	118.45 (12)	C19—C18—H18	120.00
C15—C16—C17	121.38 (13)	C18—C19—H19	120.00
C16—C17—C18	119.72 (14)	C20—C19—H19	120.00
C17—C18—C19	119.41 (13)	C15—C20—H20	120.00
C18—C19—C20	120.92 (14)	C19—C20—H20	120.00
C15—C20—C19	120.11 (14)	C21—C22—H22	120.00
C11—C21—C22	118.45 (13)	C23—C22—H22	120.00
C11—C21—C26	122.14 (13)	C22—C23—H23	120.00
C22—C21—C26	119.31 (14)	C24—C23—H23	120.00
C21—C22—C23	120.26 (14)	C23—C24—H24	120.00
C22—C23—C24	120.17 (15)	C25—C24—H24	120.00
C23—C24—C25	119.91 (15)	C24—C25—H25	120.00
C24—C25—C26	120.24 (14)	C26—C25—H25	120.00
C21—C26—C25	120.08 (14)	C21—C26—H26	120.00
C2—C1—H1	120.00	C25—C26—H26	120.00
C6—C1—H1	120.00		
			
C8—O1—C4—C3	−171.98 (13)	C5—C6—C9—N2	−143.78 (14)
C8—O1—C4—C5	6.6 (2)	C1—C6—C9—N1	−143.64 (15)
C7—O2—C3—C4	176.30 (13)	C5—C6—C9—N1	33.3 (2)
C7—O2—C3—C2	−4.1 (2)	N1—C10—C11—N2	0.70 (15)
C10—N1—C9—N2	−0.18 (15)	C11—C10—C15—C20	28.2 (2)
C9—N1—C10—C11	−0.34 (15)	N1—C10—C15—C20	−152.15 (13)
C10—N1—C9—C6	−177.67 (12)	C11—C10—C15—C16	−154.50 (15)
C9—N1—C10—C15	179.97 (11)	C15—C10—C11—C21	5.1 (2)
C12—N2—C11—C21	−8.3 (2)	N1—C10—C15—C16	25.13 (19)
C11—N2—C9—C6	178.00 (13)	N1—C10—C11—C21	−174.60 (13)
C9—N2—C11—C21	174.87 (13)	C15—C10—C11—N2	−179.65 (13)
C12—N2—C11—C10	176.06 (12)	N2—C11—C21—C22	−109.09 (16)
C11—N2—C9—N1	0.61 (15)	C10—C11—C21—C26	−110.82 (18)
C12—N2—C9—N1	−176.05 (13)	N2—C11—C21—C26	74.64 (18)
C11—N2—C12—C13	81.73 (17)	C10—C11—C21—C22	65.5 (2)
C9—N2—C12—C13	−102.15 (17)	N2—C12—C13—C14	4.5 (2)
C9—N2—C11—C10	−0.78 (15)	C10—C15—C16—C17	−176.38 (13)
C12—N2—C9—C6	1.3 (2)	C20—C15—C16—C17	1.0 (2)
C2—C1—C6—C9	178.37 (14)	C10—C15—C20—C19	177.46 (13)
C2—C1—C6—C5	1.4 (2)	C16—C15—C20—C19	0.2 (2)
C6—C1—C2—C3	−0.3 (2)	C15—C16—C17—C18	−1.5 (2)
C1—C2—C3—C4	−1.6 (2)	C16—C17—C18—C19	0.9 (2)
C1—C2—C3—O2	178.77 (14)	C17—C18—C19—C20	0.3 (2)
C2—C3—C4—C5	2.4 (2)	C18—C19—C20—C15	−0.8 (2)
O2—C3—C4—O1	0.68 (19)	C11—C21—C22—C23	−174.93 (13)
O2—C3—C4—C5	−177.97 (13)	C26—C21—C22—C23	1.5 (2)
C2—C3—C4—O1	−178.96 (13)	C11—C21—C26—C25	175.78 (13)
C3—C4—C5—C6	−1.2 (2)	C22—C21—C26—C25	−0.5 (2)
O1—C4—C5—C6	−179.74 (13)	C21—C22—C23—C24	−1.2 (2)
C4—C5—C6—C1	−0.7 (2)	C22—C23—C24—C25	0.0 (2)
C4—C5—C6—C9	−177.80 (13)	C23—C24—C25—C26	1.0 (2)
C1—C6—C9—N2	39.2 (2)	C24—C25—C26—C21	−0.8 (2)

### Hydrogen-bond geometry (Å, º)

Cg3 is a centroid of the C15–C20 phenyl ring.

**Table:**

*D*—H···*A*	*D*—H	H···*A*	*D*···*A*	*D*—H···*A*
C7—H9*B*···O1^i^	0.96	2.57	3.515 (2)	170
C14—H14*A*···N2	0.93	2.53	2.859 (2)	101
C8—H8*B*···*Cg*3^ii^	0.96	2.98	3.8316 (17)	149

Symmetry codes: (i) −*x*+1, −*y*+2, −*z*; (ii) *x*, *y*+1, *z*.
